# Effects of the Edible Microalga *Chlorella* on Gut Microbiota and on Brain Health: Current Evidence and Emerging Links

**DOI:** 10.3390/nu18122014

**Published:** 2026-06-21

**Authors:** Olga Felip, Iker García, Garoa Santocildes, Joan Ramon Torrella, Ginés Viscor, Josep Lluis Torres, Sara Ramos-Romero

**Affiliations:** 1 Physiology Section, Department of Cell Biology, Physiology and Immunology, Faculty of Biology, University of Barcelona, 08028 Barcelona, Spain; olga.maria.felip@upc.edu (O.F.); gsantocildes@ub.edu (G.S.); jtorrella@ub.edu (J.R.T.); gviscor@ub.edu (G.V.); sara.ramosromero@ub.edu (S.R.-R.); 2 Department of Agri-Food Engineering and Biotechnology (DEAB), Barcelona School of Agri-Food and Biosystems Engineering (EEABB), Universitat Politècnica de Catalunya-BarcelonaTech (UPC), Esteve Terradas, 8, 08860 Castelldefels, Spain; 3 Nutrition & Food Safety Research Institute (INSA-UB), Maria de Maeztu Unit of Excellence, 08921 Santa Coloma de Gramanet, Spain; joseplluis.torres@iqac.csic.es; 4 Department of Biological Chemistry, Institute of Advanced Chemistry of Catalonia (IQAC-CSIC), 08034 Barcelona, Spain

**Keywords:** dietary supplements, gut–brain axis, microalgae, neuroprotection, functional food

## Abstract

**Background**: *Chlorella*, a unicellular green alga, is currently one of the most popular algae supplements due to its high content of bioactive compounds. *Chlorella*’s wide range of macro- and micronutrients, including chlorophyll compounds and carotenoids, has been suggested to influence various disorders related to the digestive and nervous systems. This review’s primary purpose was to critically analyze the effects of *Chlorella* intake on gut microbiota and brain function. **Methods**: The authors conducted a systematic review with narrative synthesis of peer-reviewed articles written in English and published in PubMed, Web of Science, and Scopus spanning the years 2009 to 2026 (PROSPERO registration number CRD42024527705). The search protocol was performed following PRISMA guidelines. Primary outcomes encompassed physiological variables, such as gut microbial composition, short-chain fatty acids, brain-derived neurotrophic factor, and hippocampal cell density. Secondary outcomes were assessed through neurobehavioral tests and psychological questionnaires. **Results**: Out of the 1333 articles identified, 47 studies were deemed eligible, and 21 met the predefined criteria, subsequently incorporated into this systematic review. In total, 10 articles documented interventions involving *Chlorella* and their effects on the gut microbiota, whereas 11 articles investigated several variables pertinent to brain function. Most of the studies included were conducted in animal models, with only a limited number of human trials. Nineteen studies (90%), predominantly preclinical, reported positive associations between *Chlorella* consumption, gut microbiota modulation, and physiological or neurobehavioral markers related to the gut–brain axis. **Conclusions**: *Chlorella* consumption may modulate gut microbiota composition and function, potentially influencing brain-related processes. However, the available literature lacks studies simultaneously addressing both gut microbiota and brain health parameters limiting the understanding of the underlying physiological mechanisms.

## 1. Introduction

*Chlorella* is a spherical to ellipsoidal unicellular green alga, ranging from 2 to 10 µm in diameter, found in both marine and freshwater environments, and classified within the class Chlorophyceae [1,2,3,4]. It is commercialized and used worldwide, particularly in Asia, as a food source, dietary supplement, and alternative medicine [2,3,5]. The cultivation of *Chlorella* in specialized facilities represents a novel approach to CO_2_ fixation. These cultures effectively sequester CO_2_ while requiring minimal land area [4] and can be postulated as an alternative source of plant-derived protein.

*Chlorella* is composed of a wide range of biologically active substances [6], including several micro- and macronutrients (Table 1), such as carbohydrates, proteins, nucleic acids, essential amino acids, fatty acids, vitamins, minerals, and dietary fiber [3,7,8,9,10]. It is also rich in chlorophyll and carotenoids, including β-carotene and lutein, as well as phenolic compounds (flavonoids) [3,7,10]. Among these, protein is the predominant nutrient in *Chlorella* cells, comprising approximately 51–58% of their dry weight [10]. However, the biochemical profile of *Chlorella* varies among different species and depends on the cultivation conditions [4,10].

The biological responses to the *Chlorella* administration are primarily attributed to its antioxidant and anti-inflammatory components. These key physiological effects support additional health-related functions, including anti-hypertensive and anti-atherosclerotic actions, as well as the reduction in hyperglycemia and hypercholesterolemia [2,3,5,9]. The various macro- and micronutrients found in *Chlorella* may play a crucial role in maintaining the balance between the gut environment and its microbiota [5]. The gut microbiota is a complex community of microorganisms, including bacteria, viruses, fungi, archaea, and protozoa, that inhabit the mammalian gut [12,13,14,15]. The composition and activity of the gut microbiota are highly dynamic and influenced by various external factors, including physical activity, dietary habits, chronological aging, environmental conditions, and psychological stress [12,15,16]. The host’s lifestyle influences the gut microbiota, and the gut microbiota in turn has a direct impact on various physiological processes within the host. Therefore, harmonious interaction between the host and its microbiota is essential for gut and overall body health preservation [13,14].

Under physiological conditions, a balanced gut microbiota contributes to nutrient digestion and absorption, vitamin synthesis, energy homeostasis, and the development and maturation of the host immune system [13,14,15,16]. This balance is maintained by compounds such as short-chain fatty acids (SCFAs), lipopolysaccharides (LPS), and bile acids [17]. Through these compounds, the gut microbiota influences multiple host organs, including the central nervous system [13,14,15,16]. The gut microbiota is involved in maintaining the structural integrity of the blood–brain barrier as well as in central nervous system (CNS) neurogenesis, development, and neurotransmission [18]. Neurons, metabolites produced by the gut microbiota, and components of the innate immune system collectively mediate bidirectional communication between the gut microbiota and the brain, ultimately contributing to the maintenance of the host’s neurological health [14,18,19]. In contrast, dysbiosis of the gut microbiota has been observed in numerous neuropathological conditions, including depression, chronic stress, Alzheimer’s disease (AD), and Parkinson’s disease (PD) [12,14].

This review aims to summarize and critically evaluate previous research investigating the impact of *Chlorella* consumption on gut microbiota and on the brain function. Specifically, the research question guiding this review is: Does *Chlorella* supplementation, compared to placebo or no treatment, affect gut microbiota–brain axis outcomes in humans and rodents? This complex bidirectional communication system is essential for maintaining homeostasis within the gastrointestinal tract through interactions between the gut microbial community and the central nervous system [15]. This network of physiological connections includes the autonomic and enteric nervous systems, the vagus nerve, the hypothalamic–pituitary–adrenal (HPA) axis, the neuroendocrine system, the immune system, and various metabolic pathways [15,16,18,19].

## 2. Materials and Methods

### 2.1. Review Design and Search Strategy

An a priori search was conducted in the PROSPERO database Systematic Reviews to identify whether the topic of the current review had been previously explored. No registered protocols were found; hence, the present review was registered as a novel research question (registration number: CRD42024527705). This systematic review with narrative synthesis was conducted in accordance with the PRISMA (Preferred Reporting Items for Systematic Reviews and Meta-Analyses) guidelines for systematic reviews [20]. Three electronic databases (MEDLINE via PubMed, ISI Web of Science, and Scopus) were systematically searched for relevant studies from 1 January 2009 to 30 April 2026 to capture the most recent advances in the field. The search was conducted on 4 May 2026. The following terms were used: ((chlorella) AND (microbiota OR gut microbiota OR gut bacteria OR bacterial metabolites OR short-chain fatty acids OR SCFAs OR brain OR serotonin OR tryptophan OR dopamine OR neuroprotection OR neurodegenerative OR cognitive OR mental OR brain–gut axis OR gut–brain axis)). In all three databases, all fields were considered.

### 2.2. Eligibility Criteria

Peer-reviewed original articles in the English language were included; book chapters, hypotheses and conference publications, letters, oral presentations, and review articles were excluded. The study populations aimed for this review were healthy or unhealthy (any type of associated or unassociated disease) adult humans, rats, or mice, while other animal species or in vitro studies were excluded. All observational, experimental, and randomized controlled studies that assessed the effects of any *Chlorella* preparation on the gut microbiota and brain function-related characteristics (as a primary or secondary outcome) compared to placebo and those assessing the *Chlorella* effects as an adjunctive therapy were included in this review, while biomass and industrial studies were excluded. Studies with deficient data on patients, control groups, interventions, and outcomes were also excluded from this review. The research question was structured according to the PICO framework, separating preclinical populations (rodents) from clinical populations (humans) to account for translational heterogeneity. The PICOS criteria for the inclusion of studies are listed in Table 2.

### 2.3. Data Extraction and Analysis

Two authors (IG & OF) independently searched the databases and assessed the articles. In the primary screening of the articles, titles and abstracts were evaluated according to the inclusion and exclusion criteria. Full-text screening of potentially eligible articles was also performed independently and in duplicate by the same two reviewers. The reference lists of the relevant articles were manually screened to ensure that no eligible publications were missed. Disagreements about the eligibility of the articles were resolved by discussion with a third author (SRR).

Duplicate articles were identified and removed using the Rayyan AI, which was also used to manage the title/abstract screening. After careful review of the abstracts, articles were excluded if they contained keywords of interest but did not address the defined objective. Studies outside the selected time frame and those reporting the same findings or outdated results were also excluded.

If an article was considered eligible, the following information was extracted: first author’s name, year of publication, study location, number of participants in *Chlorella* and control groups, trial design, duration of intervention, daily dose, and type of *Chlorella*, age and sex of participants, and health status of subjects. To avoid the omission of relevant articles, the reference list of the included articles was subsequently checked.

### 2.4. Outcomes and Quality Assessment

The primary outcome of this systematic review was the effects of *Chlorella* on the physiological status of the gut microbiota and brain, assessed using validated tools and indices such as microbiota diversity, SCFAs, brain-derived neurotrophic factor, or hippocampal cell density. Secondary outcomes included other physiological variables associated with the gut microbiota and brain, such as erythrocyte phospholipid hydroperoxide (PHOOC) accumulation and neurobehavioral assessment results. Due to the considerable heterogeneity in study designs, including differences in populations (animal vs. human), *Chlorella* preparations, dosages, and health outcomes, a statistical meta-analysis was not performed. The heterogeneity across studies was also taken into account when assessing the overall strength of the evidence.

SYRCLE’s Risk-of-Bias tool [21] was used to assess the quality of the selected studies involving rodents. The Cochrane Risk-of-Bias 2.0 tool (RoB2) [22] was used to assess the quality evaluation of selected studies involving humans. Three authors (IG, OF, and GS) separately assessed the risk of bias in each included study. Disagreements were resolved in consultation with a fourth reviewer (SRR). Based on the rating across all domains, each study was given an overall rating of “high risk,” having “some concern,” or “low risk” for the RoB2, while SYRCLE’s guidelines encourage a qualitative interpretation.

## 3. Results

### 3.1. Selection and Identification of Studies

The relevant publications were selected as follows: 1. Identification of availability of the relevant sources; 2. Checks to eliminate duplication; 3. Assessment of relevance: verification that the selected study aligns with the stated objective; 4. Evaluation of the studies.

**Figure 1:**
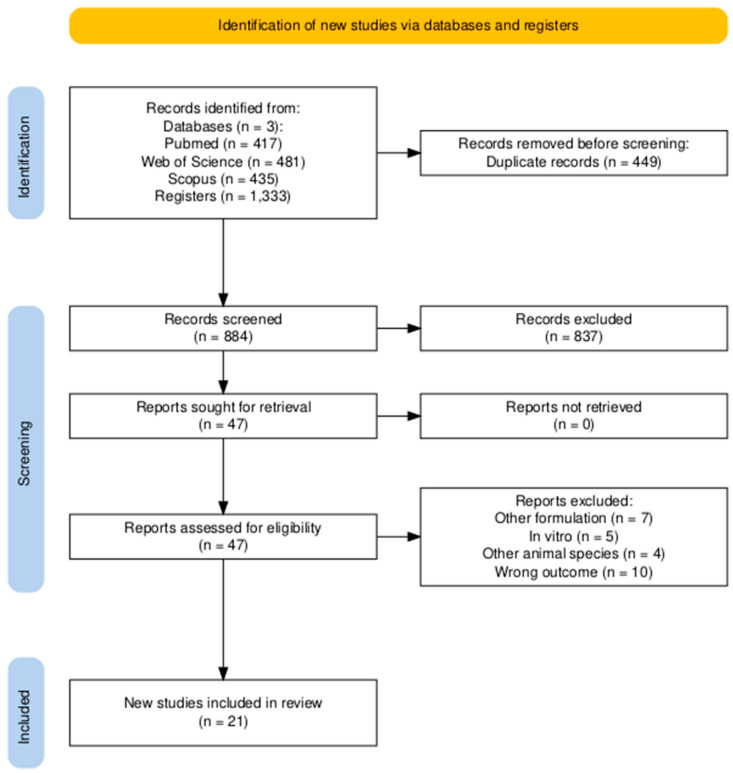
PRISMA flowchart describing the systematic literature search and study selection.

A flowchart illustrating the study selection process was generated using PRISMA2020 [23] (Figure 1). In summary, our systematic search yielded 1333 records, of which 449 duplicate records were eliminated. Of the remaining records, 837 were excluded at the title and abstract screening stage because they were irrelevant to the topic, based on studies in different animal species, were review articles, or lacked data on the parameters under consideration. Finally, 47 records were carefully revised (Table S1), and 21 records [1,2,3,6,8,24,25,26,27,28,29,30,31,32,33,34,35,36,37,38,39] were included in the review.

### 3.2. Characteristics of the Selected Studies

A total of 317 rats, 360 mice, and 177 humans were analyzed in this systematic review. Six studies involved female participants [1,2,3,29,30,31]. Three studies involved humans [2,3,31], eight included rats [6,8,25,26,30,32,36,39], and eight were carried out on mice [1,24,27,29,33,34,35,37,38]. In terms of stratification by outcome, ten studies examined the gut microbiota [2,6,24,25,26,27,28,35,37,38] and eleven focused on brain health [1,3,8,29,30,31,32,33,34,36,39]. *Chlorella* supplementation was mainly used at dosages of 30 to 400 mg/kg/day in animal models and 1 to 8 g/day in humans. The duration of the intervention ranged from 1 to 6 weeks in eight studies [2,27,29,30,33,34,36,37]; ten studies included an intervention between 6 and 12 weeks [3,6,24,25,26,28,31,35,38,39]; one study was longer (70 weeks) [1]; and two included an acute treatment (single dose) [8,32].

### 3.3. Methodological Quality and Risk Bias

**Figure 2:**
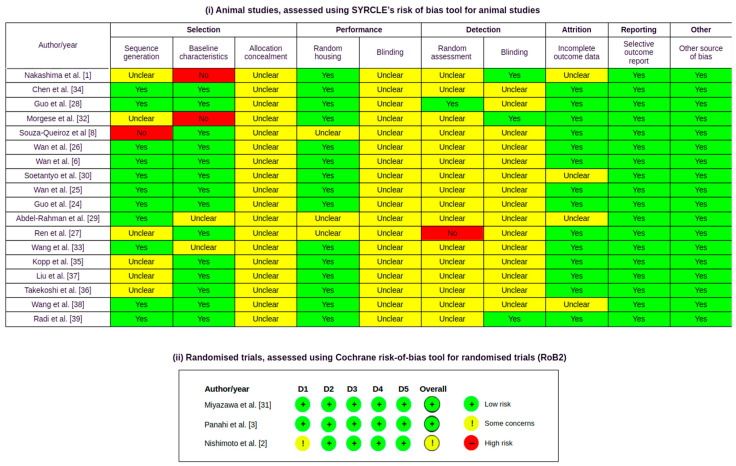
Risk of Bias assessment. D1: Bias due to randomization; D2: Bias due to deviations from intended interventions; D3: Bias due to missing data; D4: Bias due to outcome measurement; D5: Bias due to selection of the reported result. Overall: Overall risk of bias [1,2,3,6,8,24,25,26,27,28,29,30,31,32,33,34,35,36,37,38,39].

The risk of bias assessment is summarized in Figure 2. According to the quality of the studies involving humans in RoB2, two studies were assessed as having a low risk of bias [3,31], while one study had some concerns about bias [2]. Regarding SYRCLE’s, the studies included in this review had low risk for the Selection, Attrition, and Reporting bias, while Performance and Detection showed unclear or high risk of bias.

Given that most included studies were conducted in animal models, a formal GRADE assessment was not applied; we therefore appraised the evidence narratively, weighing study design, risk of bias, consistency of findings, and translational distance to humans. Of the 21 included studies, 90% reported beneficial effects of *Chlorella* on gut microbiota or brain-related outcomes, consistent across both rodent and human data. Selection, Attrition, and Reporting bias were generally low, whereas Performance and Detection bias were unclear or high in most animal studies. Publication bias cannot be excluded due to the predominance of positive findings across preclinical studies, combined with the small number of human trials, which raises the possibility of selective reporting.

Taken together, the evidence is consistent, with some biological plausibility to the role of *Chlorella* in modulating the gut microbiota–brain axis. Nevertheless, heterogeneity in preparations and outcome measures makes cross-study comparison difficult, and well-powered RCTs in humans are needed before clinical inferences can be drawn.

### 3.4. Chlorella’s Impact on the Gut Microbiota

The effects of *Chlorella* intake on the gut microbiota have been investigated in 10 studies, 9 of which were conducted in animal models (Table 3) and 1 in human participants [2]. Some studies employed *Chlorella* extracts, such as ethanolic extracts [6,26] or specific isolated components, including various polysaccharides [24,25], while others used the whole *Chlorella* formulation [2,27,28,35,37,38]. Most trials assessing the impact of *Chlorella* supplementation on the gut microbiota utilized methodologies such as metabolomic analysis, 16S rRNA sequencing of cecal content, or correlation analyses between clinical biochemical indices and cecal microbiota composition.

Our review indicates that while there is inherent heterogeneity, the core methodologies for microbiota determination are fundamentally comparable as they rely on high-throughput sequencing rather than traditional culture-based or low-resolution molecular methods. The chosen studies consistently utilized high-throughput sequencing to determine taxonomic profiles. The majority employed the Illumina MiSeq platform [2,24,27,28,35] while others used the Ion S5 or IonS5™XL systems [6,25]. Regarding genomic targets, most studies focused on the V3–V4 hypervariable regions [6,24,25,27,35], which is widely considered the gold standard for providing a balanced view of gut community structure. Variations include targeting the V4 region [28], V1–V2 [2], or V4–V5 [24,38]. Furthermore, a recent study incorporated macrogenomics (shotgun metagenomics) via DNA assembly (MEGAHIT) [37], allowing for species-level and functional annotation beyond the resolution of 16S sequencing. Functional activity was assessed through Gas Chromatography (GC) with Flame Ionization Detection (FID) for targeted SCFA analysis [24,38]. Advanced studies complemented this with untargeted metabolomics using LC-MS (Triple TOF) or CE-TOFMS to capture broader dicarboxylic acid and lipid-like metabolite profiles [2,37].

The administration of *C. pyrenoidosa* in male mice and rats subjected to a high-fat diet (HFD) enhances the α-diversity of the gut microbiome, reflecting increased bacterial species richness, and restores β-diversity, indicating improvements in community structure [24,25,26]. Several studies reported a decrease in the Firmicutes/Bacteroidetes ratio, a marker of micro-ecological imbalance in the gut [6,24,26,28]. Nishimoto et al. [2] observed increased levels of various dicarboxylic acids in human fecal samples and found correlations between the genus *Ruminiclostridium*_9 (phylum Firmicutes) and *Butyricimonas* (phylum Bacteroidetes) with elevated butyrate and propionate, recognized indicators of optimal digestive function. Additionally, evidence from a mouse model [38] and the sole human study available to date [2] indicate that individuals with low baseline fecal propionate exhibited increased levels following *Chlorella* consumption. *C. pyrenoidosa* restored HFD-induced dysbiosis and modulated gut bacterial enzymes, transcription factors, and metabolic pathways (AMPKα, ACC, HMG-CoA, CPT1, SREBP-1c, PPARγ) involved in the metabolism of SCFAs and secondary bile acids [24,25,38]. Kopp et al. [35] demonstrated that *C. vulgaris* reduced lipopolysaccharide translocation and plasma endotoxin levels. Histopathological and biochemical analyses further revealed that *Chlorella* ingestion enhanced the metabolism of fecal total bile acids (TBAs) [25,26] and SCFAs (acetate, propionate, and butyrate) [24,38], while upregulating AMPKα and downregulating ACC, SREBP-1c, and HMG-CoA expression. Consequently, *Chlorella* supplementation may influence the host’s biological pathways via gut microbial metabolism, exerting health benefits such as anti-inflammatory effects and promoting overall well-being [38].

Distinct responses were observed among specific bacterial taxa following *Chlorella* administration. Within the phylum Bacillota, the genera *Lactobacillus* and *Turicibacter* showed either decreases [6] or increases [25], depending on the experimental conditions, whereas *Ruminococcus* [6,25,26] and *Oscillibacter* consistently increased [6]. Conversely, the populations of the genus *Blautia* [6] and *Lachnospira* [25,26] decreased after *Chlorella* administration. Within the phylum Bacteroidetes, microalgal supplementation increased the populations of the genera *Bacteroides* and *Alistipes* [26,37]. Other probiotic-associated taxa also increased, including Actinobacteria (genus *Bifidobacterium* [28]), Verrucomicrobia (genus *Akkermansia* [6,27]), and Pseudomonadota (*Parasutterella* [6]). In search of alternative antibiotics, Guo et al. [28] studied the effect of a preparation of *C. ellipsoidea* expressing neutrophil peptide 1 (NP-1) and reported increased populations of Gram-negative Clostridia and Gram-positive lactic acid bacteria within the Actinobacteria and Bacilli classes, key modulators of gut microbiota composition.

Overall, these studies consistently observed favorable modifications in gut microbiota composition, effectively restoring microbial balance and potentially influencing disorders such as obesity and hyperlipidemia [24,25,26], hypercholesterolemia [24,25,26], or hyperglycemia [2,6,27].

### 3.5. Chlorella’s Impact on the Brain

Within the context of this review, we defined “Brain Health” as the preservation of neurological integrity, encompassing biochemical balance, structural preservation, and functional/behavioral performance. The effects of *Chlorella* consumption on brain function and psychological disorders were examined in 11 studies, of which 9 were conducted in animal models (Table 4) and 2 in human participants (Table 5). One of these studies examined extracted *C. pyrenoidosa* peptides (CPPs) [33], while another focused on a polysaccharide [34]. The remaining nine utilized the whole *Chlorella* biomass [1,3,8,29,30,31,32,36,39]. The physiological and behavioral variables associated with brain function included serotonin, dopamine, and brain-derived neurotrophic factor (BDNF) levels, responses in the forced swimming test (FST), the Morris water maze (MWM) test, and chronic unpredictable mild stress (CUMS), among others.

*Chlorella* supplementation has been shown to alleviate both the physical and cognitive manifestations associated with depression [3,8,30], as well as behavioral variables linked to anxiety [3,29] in both humans and rodents, suggesting a potential antidepressant role. These findings are consistent with the observed reduction in stress-induced HPA axis activation, as indicated by a decreased adrenocorticotropic hormone (ACTH) response [8]. This may provide a mechanistic link between *Chlorella* and the mitigation of anxiety and depressive symptoms.

Supplementation with *Chlorella* has also been reported to enhance short-term memory [32,33,39] and cognitive function [1,34]. *Chlorella* intake demonstrated efficacy in ameliorating bradykinesia and preventing depletion of striatal dopamine and its metabolites, thereby increasing tyrosine hydroxylase levels in murine models of PD [34]. Similarly, Wang et al. [33] reported improvements in spatial cognition and learning memory, as well as restoration of cellular loss in the CA1 and CA3 regions of the hippocampus in Aβ1–42-induced AD mouse models, although activation of the BDNF–TrkB–CREB signaling pathway in the hippocampus was not observed [36]. These neuroprotective effects are further supported by Radi et al. [39], who demonstrated that Chlorella exerts strong neuroprotection in AD-induced mouse models, evidenced by reductions in p-Tau and Beta-Amyloid levels, an increase in BDNF expression, and attenuation of hippocampal degeneration. Notably, the BDNF upregulation reported by Radi et al. [39] contrasts with the absence of BDNF–TrkB–CREB pathway activation observed by Wang et al. [33], suggesting that *Chlorella* may modulate BDNF through distinct mechanisms depending on the model or treatment conditions used. Neurochemical studies revealed region-specific effects of *Chlorella*, including increased serotonin content in the hippocampus [32] and decreased number of activated astrocytes in the DAL101 brain [1]. In humans, a reduced accumulation of erythrocyte phospholipid hydroperoxide (PLOOH) [31] and improvements in depression and anxiety behavioral patterns [3] have also been described. These findings provide a potential scientific rationale for the therapeutic application of *Chlorella* in the treatment of senile dementia.

In most of the reviewed studies [1,3,8,29,30,31,32,33,34,39], administration of *Chlorella* or its components enhanced biochemical and neurobehavioral parameters in the brain. This includes modulation of inflammatory markers, which are associated with neuroinflammation, cognitive deficits, and brain tissue damage.

## 4. Discussion

This review summarizes the current evidence on the relationship between *Chlorella* consumption and both gut microbiota composition and brain function. Importantly, the existing literature is predominantly derived from preclinical studies, with relatively few human studies available, particularly those addressing gut microbiota–brain interactions.

The gut–brain axis represents a continuous, bidirectional communication network that involves direct neural pathways (vagus nerve, enteric nervous system, and spinal nerves), as well as endocrine (HPA axis and gut hormones) and immune (cytokine-mediated) signaling through systemic circulation [40]. Alterations in the relative abundance and diversity of microbiota, as well as in microbial metabolite profiles, are associated with a broad spectrum of neurological and psychiatric disorders, including PD, AD, and major depressive disorder [41,42]. Indeed, the term “psychobiotics” has recently emerged to describe microbial-based interventions targeting mental health, underscoring the growing interest in the gut–brain axis as a therapeutic target [43]. Although these physiological systems are closely interrelated, studies simultaneously addressing the role of *Chlorella* in both gut microbiota composition and brain function are lacking, which reduces the overall strength of the evidence presented in this review.

The gut microbial community is essential for maintaining normal physiological functions, with diet acting as a key modulator of the dynamic relationship between the intestinal microbiota and host health [44]. The adult gut microbiota exhibits remarkable plasticity, allowing rapid adaptation to dietary changes and supporting the diversity of human diets [45]. *Chlorella* supplementation has been shown, primarily in animal studies, to enhance the biosynthesis of microbial-derived SCFAs [24,25,38], which have been reported to reverse the behavioral and physiological effects of chronic stress in germ-free mice [46]. Increased butyrate production has been associated with the alleviation of cognitive deficits in a vascular dementia model [47]. Additionally, colonization by SCFA-producing bacteria reduced blood–brain barrier permeability, highlighting the role of SCFAs in barrier integrity [48].

Dietary *Chlorella* supplementation has been reported to reduce the Firmicutes/Bacteroidetes ratio, driven by a decrease in Firmicutes abundance and a concurrent increase in Bacteroidetes populations, as observed in both in vitro and in vivo studies [6,24,49]. Approximately 90% of bacteria in the mouse and human gut belong to the phyla Bacteroidetes and Firmicutes [50]. This ratio plays a critical role in host metabolism, as Firmicutes possess carbohydrate transporters that enhance energy absorption, while Bacteroidetes produce enzymes that facilitate the breakdown of dietary carbohydrates [51]. A lower proportion of Firmicutes and a higher proportion of Bacteroidetes have been associated with neurological and mental health conditions, including AD and cognitive impairment in elderly individuals [52,53]. Populations of other phyla, including Verrucomicrobia, Proteobacteria, Actinobacteria, Clostridia, and Mollicutes, within the intestinal tract of mice were also modified in response to *Chlorella* supplementation [52,53]. Administration of *C. pyrenoidosa* extract induced changes at lower taxonomic levels, such as increased abundance of the bacterial genera *Alistipes*, *Prevotella*, *Alloprevotella*, *Ruminococcus*, and *Parasutterella*, and the class Erysipelotrichaceae, alongside decreased abundance of *Turicibacter*, *Lachnospira*, *Lactobacillus*, and *Blautia* [6,25].

In relation to these microbiota-modulating effects, the bioactivity of *Chlorella* in any physiological system, and specifically on the gut–brain axis, strictly depends on the bioaccessibility and stability of its bioactive components during gastrointestinal transit. The rigid, microfibrillar cellulosic cell wall of *Chlorella* represents a major barrier to digestion; without adequate mechanical or enzymatic cell-wall disruption before its intake, intracellular proteins, carotenoids, and lipids remain trapped within the matrix, drastically reducing their release in the upper gastrointestinal tract. Cell disruption method can affect protein digestibility by modifying its bioaccessibility after digestion [54], and more broadly, the stability and release from the cellular matrix of other bioactive compounds along the gastrointestinal tract [55]. Supporting these results, Chen et al. [56] demonstrated that the microalgal polysaccharides are not digested in the small intestine and can therefore enter directly into the colon and be utilized by the gut microbiota, where they become available for microbial biotransformation. Consistently, Bañares et al. [57] demonstrated that *C. vulgaris* can increase SCFA production during colonic fermentation, using a sequential approach combining the INFOGEST static digestion model with in vitro colonic fermentation, which may partly explain the microbiota-modulating properties attributed to this microalga.

Beyond its effects on gut microbiota composition, a growing body of preclinical research has examined the effects of *Chlorella* on brain health, yielding significant findings such as alleviation of stress, anxiety, and depression symptoms, as well as improvements in cognitive function [8,29]. Stress can substantially disrupt the homeostasis of the gut microbiota–brain axis across different life stages [58]. Emotional stressors activate multiple brain regions, as indicated by the expression of immediate early genes such as *c*-Fos [8]. Acute administration of *C. vulgaris* the day before the swimming forced test (SFT) significantly attenuated both peripheral and central HPA axis responses following the test, demonstrating the efficacy of *Chlorella* in mitigating the effects of acute stressors on the brain [8]. Moreover, *Chlorella* exhibited a synergistic effect when combined with lion’s mane mushroom (*Hericium erinaceus*), resulting in increased time spent in the central zone during the open-field test (OFT) and reduced immobility during SFT [59]. This effect of *C. vulgaris* may be linked to its influence on tryptophan metabolism and the serotonergic system through modulation of the enteric microbiota [60]. While preclinical behavioral assays (e.g., SFT, MWM) provide valuable insights into the neurobiological effects of *Chlorella*, they cannot be extrapolated to clinical human outcomes. These models serve as proxies for underlying physiological changes rather than direct clinical equivalents of human neuropsychiatric disorders. In humans, *C. vulgaris* reduced both somatic and cognitive symptoms of depression [3], the most prevalent mental disorder and a leading cause of disability worldwide [61]. Notably, even a small amount of *Chlorella* (0.2 mL of concentrated extract) elevated BDNF levels, highlighting its potential for alleviating depressive symptoms [59]. Nevertheless, human evidence supporting these neurobehavioral effects remains limited and inconclusive.

As the prevalence of Alzheimer’s disease (AD)-related dementia is projected to triple by 2050, there is an urgent need to develop strategies to mitigate its impact [62]. Evidence for neuroprotective effects in neurodegenerative disease models is limited to animal studies, and human translational data is currently lacking. Acute oral administration of a lipid extract of *C. sorokiniana* improved memory performance in rats, accompanied by significant increases in noradrenaline and serotonin levels in the hippocampus, specifically within hilar perforant path-associated cells [32]. The hilar region plays a critical role in both spatial memory and the perception of non-spatial objects [63].

*Chlorella* supplementation also attenuated declines in spatial memory and learning in mitochondrial aldehyde dehydrogenase 2 activity-deficient transgenic mice (DAL101), as evidenced by the prevention of age-related impairments in recognition memory using the novel object recognition test [1]. Furthermore, *Chlorella* reduced the number of reactive astrocytes in the hippocampal CA1 region, thereby limiting neuronal damage in DAL101 mice [1]. Short peptides from *C. pyrenoidosa* improved spatial cognition and learning memory, with a reduced cell loss ratio in the CA1–CA3 hippocampal regions [33]. These effects may have therapeutic relevance for AD, potentially mediated by anti-inflammatory and anti-amyloid activities, as demonstrated in vitro by reductions in amyloid precursor protein and tau neurofibrillary tangles [33].

*Chlorella* contains high levels of carotenoids, including astaxanthins, which may confer neuroprotective effects against neurodegenerative disorders by inhibiting apoptosis, mitigating mitochondrial dysfunction, and reducing excessive reactive oxygen species (ROS) production [64]. The findings of this review highlight the potential of *Chlorella* intake to alleviate oxidative stress and support neuronal health in neurodegenerative conditions. *C. pyrenoidosa* demonstrated neuroprotective effects on dopaminergic neurons in vivo in the MPTP-induced neurotoxicity model of PD in mice, likely through suppression of pro-inflammatory mediators produced by activated resident microglia, thereby preventing dopaminergic cell loss in the nigrostriatal pathway [34]. These findings are primarily based on experimental models, and their translation to human neurological conditions remains to be established.

*Chlorella* is a rich source of lutein [65], a carotenoid known for its antioxidant properties that protect human erythrocytes. Supplementation with *Chlorella* reduced the accumulation of PLOOH in the erythrocyte membrane and increased plasma lutein levels in humans [31], suggesting that its consumption may help maintain normal erythrocyte function and prevent the development of senile dementia in humans. Elevated PLOOH levels in erythrocyte membranes impair gas exchange, including O_2_ delivery to the brain, which is associated with cognitive decline [66]. Previous studies have reported higher PLOOH accumulation in the erythrocytes of dementia patients, including those with AD, who exhibited excessively oxidized erythrocytes [66,67].

The intestinal barrier plays a critical role in separating the gut microbial ecosystem from the gut-associated immune system, despite their proximity [68]. Diet-induced release of immune mediators into the systemic circulation, known as metabolic endotoxemia, can trigger immune activation in various organs, including the brain [69]. Local immune activation may increase the permeability of epithelial tight junctions, further compromising the intestinal barrier [40]. This low-grade immune activation has been linked to the pathophysiology of certain forms of depression and neurodegenerative disorders, including AD and PD [40]. Exclusively demonstrated in preclinical studies, supplementation with *C. vulgaris* has been shown to enhance immune cell function by promoting lymphocyte proliferation and macrophage phagocytic activity [70]; some evidence points to beneficial effects of its polysaccharides [71], though not of its omega-3 fatty acids [72]. *C. vulgaris* may also directly influence bone marrow (myelostimulation) by inducing endogenous cytokine production [73], providing prophylaxis against post-stress myelosuppression [74]. Polysaccharides derived from *C. pyrenoidosa* act as anti-inflammatory mediators in the communication between the immune system and the brain, contributing to the reduction of PD-associated symptoms [34].

In humans, although evidence is limited to a few studies, *Chlorella* consumption has been associated with antioxidant [7], anti-inflammatory [75], and immunostimulatory effects, resulting in increases in cytokine production (IFN-γ, IL-1α, TNF-α) and natural killer (NK) cell activity [9]. These findings indicate that *Chlorella* could enhance systemic physiology by modulating the immune system. Notably, very few human studies have simultaneously evaluated gut microbiota and neurological outcomes.

The mechanisms underlying the neuroprotective effects of microalgae and their bioactive compounds remain incompletely understood [76]. Neural connections between sensory cells of the gut epithelium and the nervous system facilitate bidirectional signaling, potentially linking gut microbiota and brain function [77]. Microalgal compounds exert neuroprotective effects through antioxidant activity, enhancement of cholinergic function, and inhibition of β-amyloid aggregation and neuronal damage in AD brains [76]. These mechanisms underscore the central role of oxidative stress in the onset and progression of neurodegenerative disorders, including AD. Among carotenoids derived from *Chlorella*, astaxanthin has been shown to efficiently cross the blood–brain barrier and act as a potent free radical scavenger [78]. *Chlorella* has also demonstrated efficacy in mitigating oxidative brain damage induced by lead exposure in rats, enhancing enzymatic antioxidants (superoxide dismutase, catalase, glutathione peroxidase) and non-enzymatic antioxidants (glutathione), while reducing malondialdehyde levels [79]. Similarly, the free radical scavenging properties of *C. vulgaris* were confirmed in albino rats subjected to naphthalene-induced oxidative stress [80].

A critical factor in interpreting the neurological effects of *Chlorella* is the heterogeneity of the preparations used across studies. Whole biomass interventions primarily exert their effects through the synergistic action of fiber and intact nutrients, promoting prebiotic-like shifts in the microbiota [2,35,38]. In contrast, aqueous extracts (rich in *Chlorella* Growth Factor and peptides) and ethanolic extracts (concentrating lipophilic antioxidants like lutein) bypass the digestive challenge of the microalga’s rigid cellulosic wall [6,30,39]. These concentrated fractions often yield more immediate biochemical changes in the CNS (e.g., neurotransmitter levels or oxidative stress markers) compared to whole powder [32,39]. For instance, polysaccharide-rich fractions have been specifically linked to the activation of intestinal immune receptors [33,34], whereas peptides and lipids may exert more direct neuroprotective effects [32,33,36].

Dose comparability across species also warrants consideration. Animal studies administered *Chlorella* at 300–600 mg/kg/day or as 15% of the total diet, which, when converted using allometric scaling, yields human-equivalent doses broadly consistent with the 1.8–8 g/day range used in clinical studies. Although direct extrapolation is constrained by interspecies differences in gut physiology and metabolism, these figures suggest that preclinical doses may be feasible for dietary use in humans. Standardization of dosing protocols and reporting of preparation type across future trials will be essential to establish effective dose ranges for clinical application.

In tandem with dosage standardization, the analytical methodologies used to assess microbiota composition across the compiled literature represent another critical factor that warrants careful consideration. The reviewed studies rely on fundamentally similar analytical platforms and consistently utilize high-throughput sequencing to determine taxonomic profiles. However, variations in the targeted hypervariable regions (e.g., V3–V4 vs. V1–V2) and DNA extraction kits are known to introduce taxometric biases, particularly affecting the amplification of rigid Gram-positive taxa like certain Bacillota (Firmicutes). While broad shifts (e.g., the Firmicutes/Bacteroidetes ratio) remain comparable across platforms, species-level identification is generally more robust in studies utilizing macrogenomics [37]. Also, several studies rely on computational tools like PICRUSt, PICRUSt2, or Tax4Fun to predict metabolic pathways (e.g., SCFA biosynthesis or bile acid metabolism) based on 16S data [24,38]. While these provide high-level insights, the interpretation is most reliable when corroborated by direct metabolite measurements via GC or LC-MS, as seen in the more recent literature included in this review [37,38].

Considerable heterogeneity across studies—including differences in populations (human vs. animal), *Chlorella* preparations, dosages, and healthy outcome measures—undermines the reliability and generalizability of the findings. This variability makes direct comparisons and prevents any firm conclusions regarding cause–effect relationships. In particular, the predominance of preclinical models combined with the scarcity of human trials, further weakens the overall body of the evidence.

### Future Directions

Dietary interventions aimed at modulating the gut microbiome represent a highly promising avenue for enhancing mental health. However, to transition from anecdotal evidence to clinical application, future research on *Chlorella* must prioritize the standardization of biomass production. Variability in cultivation conditions (autotrophic vs. heterotrophic) and cell-wall disruption methods substantially affect the bioavailability of neuroactive compounds, underscoring the need for a unified quality-control framework. Furthermore, it is essential to validate specific gut–brain biomarkers, such as the quantification of fecal SCFAs and plasma levels of tryptophan and its metabolites (kynurenine pathway), to establish a causal link between *Chlorella* intake and CNS modulation. Finally, while preclinical data are encouraging, large-scale, long-term (e.g., >6 months) double-blind clinical trials are necessary. These studies should focus on diverse cohorts to explore how baseline microbiota composition (enterotypes) influences the efficacy of *Chlorella* as a functional food for maintaining cognitive function and preventing neuroinflammation. Key Research Priorities:

- Priority 1: Development of standardized *Chlorella* processing techniques to ensure consistent bioactive profiles.
- Priority 2: Longitudinal monitoring of neuro-inflammatory markers (IL-6, TNF-α) in human intervention trials.
- Priority 3: Assessment of the synergistic effects between *Chlorella* polysaccharides and specific probiotic strains.

Despite these limitations, an increasing body of evidence in vivo supports the role of the gut microbiome as a contributing factor in various CNS processes and in the development of psychological and neuropsychiatric disorders. In this context, *Chlorella*, like other microalgae, can be considered a valuable source of diverse bioactive compounds that may contribute to brain health through both direct effects on the CNS and indirect actions mediated via modulation of the gut microbiota. Beyond its established potential as an alternative dietary source of high-quality proteins, *Chlorella* warrants further investigation into the health-promoting properties of its bioactive components. Further research into these compounds could position *Chlorella*, and potentially other microalgae, as important functional foods for supporting cognitive function and overall brain health.

## 5. Conclusions

Current evidence suggests that *Chlorella* has the potential to beneficially influence both gut and brain health through its association with modulation of the gut microbiota, immune responses, and neuroprotective mechanisms. However, the current body of literature is largely based on animal studies, with only a few human trials. Therefore, the clinical relevance of these findings should be interpreted with caution.

Current data suggest that *Chlorella* supplementation correlates with enhanced microbial diversity and short-chain fatty acid production while reducing lipopolysaccharide levels. These microbial changes could be associated with improved intestinal barrier integrity and reduce inflammation, which in turn are hypothesized to influence gut–brain communication via immune, endocrine, and vagal signaling pathways.

Further well-designed clinical and mechanistic studies are needed to elucidate these pathways and to decouple causal mechanisms from these observed links between *Chlorella*-induced microbiota modulation and neurological outcomes. Integrative approaches combining microbiome, metabolomic, and neurofunctional assessments will be essential to determining whether these correlative interactions translate into meaningful health outcomes in humans.

## Figures and Tables

**Table 1:** Biochemical composition of *Chlorella* [3,10,11].

*Dietary Components*	
**Amino Acids**	
*Essential*	Isoleucine, Leucine, Lysine, Methionine, Phenylalanine, Threonine, Tryptophan, Valine, Histidine
*Non-essential*	Tyrosine, Cystine, Aspartic Acid, Serine, Glutamic Acid, Proline, Glycine, Alanine, Arginine
**Fatty acids (FAs)**	Saturated FA, Monounsaturated FA, *n*-3 and *n*-6 Polyunsaturated FA
**Carbohydrates**	β-1-3-Glucans, α-Glucans, Dietary Fibers
**Vitamins**	B1, B2, B3, B5, B6, B12, C, D2, E, K, Niacin, Folate, Biotin, Pantothenic Acid
**Minerals**	Sodium, Iron, Calcium, Potassium, Magnesium, Zinc, Copper, Phosphorus, Manganese
**Pigments**	Chlorophylls, Carotenoids, Lutein

**Table 2:** PICOS criteria for inclusion of studies.

*Parameter*	*Criterion*
Population	Healthy and unhealthy female and male humans and rodents
Intervention	Specific dietary changes, or the use of nutritional or dietary supplements (in pill, tablet, powder, or liquid form) All timings, frequencies, and dosages of treatment are eligible for inclusion
Comparator	Placebo controls, sham-treated humans/animals, vehicle-treated animals, and humans/animals undergoing no treatment at all
Outcomes	The primary outcomes are the physiological effects on the gut microbiota–brain axis (e.g., short-fatty acids, butyrate, folate, brain-derived neurotrophic factor, dopamine, etc.). Secondary outcomes related to the gut microbiota–brain axis, such as erythrocyte phospholipid hydroperoxide (PHOOC) accumulation, and psychological questionnaires will be considered
Study design	Observational, experimental, and randomized controlled trial (of any design)

**Table 3:** Preclinical studies examining the effects of *Chlorella* administration on gut microbiota.

Author (Year)	Subjects	*Chlorella* Formulae	Study Design	Microbiota-Related Outcomes
Guo et al. [28]	Sprague Dawley Rat ♂ (n = 70)	- *C. ellipsoidea* - NP-1 transgenic *C. ellipsoidea*	Dosage: 1.25, 2.5, 5% Diet: Standard Duration: 8 weeks	*C. ellipsoidea* (5%): ↑ Firmicutes, Actinobacteria, Clostridia ↓ Bacteroidetes, Verrucomicrobiota, Bacilli, Lactobacillales NP-1 *C. elliposoidea* (5%): ↑ Firmicutes, Actinobacteria, Bacilli ↓ Bacteroidetes, Verrucomicrobia, Proteobacteria, Lentisphaerae, Spirochaetes, Fibrobacteres, Elusimicrobia
Wan et al. [26]	Wistar Rat ♂ (n = 32)	*C. pyrenoidosa* 55% ethanol extract	Dosage: 150 mg/kg (Standard), 300 mg/kg (High-Fat) Diets: Standard/High-Fat Duration: 8 weeks	*C. pyrenoidosa* (300 mg/kg) in high-fat diet: ↑ *Alistipes*, *Bacteroides*, and *Ruminococcus*_1, *Alloprevotella*, *Ruminococcacaeae*_UCG-010 ↓ *Lachnospira*, *Turicibacter*, and *Ruminococcus_gauvreauii_group* *C. pyrenoidosa* (both dosage) in standard and high-fat diets: ↑ Fecal total bile acids levels
Wan et al. [6]	Rat ♂ (n = 40)	- *C. pyrenoidosa* 55% etanol extract - *C. pyrenoidosa* water extract	Dosage: 150 mg/kg Diets: Standard/High-Fat High-Sucrose Duration: 8 weeks	*C. pyrenoidosa* (both extracts) in high-fat high-sucrose diet: ↑ Bacteroidetes, Verrucomicrobia, ↓ Actinobacteria and Firmicutes/Bacteroidetes ratio *C. pyrenoidosa* water extract in high-fat high-sucrose diet: ↑ *Ruminococcus*, *Akkermansia*, *Parasutterella*, Erysipelotrichaceae, and *Oscillibacter* ↓ *Lactobacillus*, Ruminococcaceae, *Turicibacter*, and *Blautia*
Wan et al. [25]	Wistar Rat ♂ (n = 40)	*C. pyrenoidosa* polysaccharide fraction	Dosage: 150 mg/kg (Standard) and 300 mg/kg (High-Fat) Diets: Standard/High-Fat Duration: 8 weeks	*C. pyrenoidosa* (both dosage) in standard and high-fat diets: ↑ *Turicibacter*, *Lactobacillus*, *Ruminococcus*_1, *Coprococcus* and *Ruminiclostridium*_5 ↓ *Lachnospira* and *Ruminococcus_gauvreauii_group* ↑ Caecal acetic and butyric levels ↑ Fecal total bile acids levels
Guo et al. [24]	C57BL/6 Mice ♂ (n = 40)	*C. pyrenoidosa* polysaccharide fraction	Dosage: 400 mg/kg/day Diets: Low-Fat/High-Fat Duration: 10 weeks	↑ α-diversity and restore β-diversity ↑ Bacteroidetes, Clostridia ↓ Firmicutes/Bacteroidetes ratio, Actinobacteria and Verrucomicrobia, Erysipelotrichia SCFAs: ↑ Acetate, Propionate, and Butyrate
Ren et al. [27]	db/db Mice ♂ (n = 10)	*C. vulgaris*	Dosage: 366.5 mg/kg/day Diet: Standard Duration: 30 days	↑ *Akkermansia* ↓ Bacterial diversity
Kopp et al. [35]	Mice ♂ (n = 64)	*C. vulgaris*	Dosage: 15% of the diet Diets: Standard/Western Style Duration: 12 weeks	*C. vulgaris* in Standard diet: =Bacteroidetes, *Clostridium IV*, *Olsenella*, *Flavonifractor* ↓ Translocation of lipopolysaccharide *C. vulgaris* in Western-style diet: =Bacteroidetes, *Clostridium IV*, *Olsenella*, *Flavonifractor* ↓ Plasma endotoxin, *Clostridium* cluster XIVa, Translocation of lipopolysaccharide
Liu et al. [37]	C57BL/6J Mice ♂ (n = 16)	*C. pyrenoidosa* peptide (SISISVAGGGR, T1)	Dosage: 600 mg/kg/day Diets: Standard/High-Fat Duration: 5 weeks	↑ *Bacteroides*, *Parabacteroides*, *Muribaculum*, *Prevotella*, *Duncaniella*, *Lactobacillus*, *Alistipes* ↓ *Ruminococcus*, *Acetatifactor*, and *Dorea* Reversed the high-fat diet-induced gut microbiota dysbiosis ↑ DL-arginine, *N*-stearoyl GABA ↓ 7α,24(S)-dihydroxy-4-cholesten-3-one and hexadecanedioic acid
Wang et al. [38]	ICR Mice ♂ (n = 50)	*C. pyrenoidosa*	Dosage: 0.8 g/kg, 4.10 g/kg Diet: Standard Duration: 12 weeks	*C. vulgaris* (4.10 g/kg): =α-diversity ↑ Lactobacillaceae and Muribaculaceae ↓ Erysipelotrichaceae and Staphylococcaceae SCFAs: ↑ Acetate and propionate

↑: increased; ↓: reduced.

**Table 4:** Preclinical studies examining the effects of *Chlorella* administration on the brain.

Author (Year)	Subjects	*Chlorella* Formulae	Study Design	Brain-Related Outcomes
Morgese et al. [32]	Wistar Rat ♂ (n = not specified)	*C. sorokiniana* extract	Dosage: 30 mg/kg Diet: Standard Duration: Single dose	Novel Object Recognition test: ↑ Time spent exploring the novel object Elevated Plus Maze locomotory test: =total exploratory activity ↑ Hippocampal serotonin and noradrenaline content =Serotonin and noradrenaline content in the prefrontal cortex and striatum
Souza-Queiroz et al. [8]	SD Rat ♂ (n = 62)	Dried *C. vulgaris*, prepared in distilled water	Dosage: 50, 200 mg/kg Diet: Standard Duration: Single dose	*C. pyrenoidosa* (200 mg/kg): ↓ ACTH levels ↓ hnCRF levels in the hypothalamus ↓ *c*-fos mRNA levels in the prefrontal cortex, hypothalamus, dorsal raphe, and focus coeruleus
Soetantyo et al. [30]	Wistar Rat ♀ (n = 25)	- Cultivated *C. vulgaris* extract - Commercial *C. vulgaris* extract	Dosage: 360 mg/kg Diet: Standard Duration: 14 days after stress induction	Both extracts: Forced Swimming Test: ↓ Immobile duration Open Field Test: ↑ Roaming behavior
Takekoshi et al. [36]	SD Rat ♂ (n = 20)	*C. pyrenoidosa* powder	Dosage: 200 mg/day Diet: Standard Duration: 1 week	BDNF signaling-related protein expression and phosphorylation: =Hippocampal BDNF expression =Phosphorylation of TrkB =Phosphorylation of CREB =Glutamate receptor expression
Radi et al. [39]	Albino Rat ♂ (n = 28)	- *C. vulgaris* - *C. vulgaris*-loaded niosome	Dosage: 100 mg/kg Diet: Standard Duration: 60 days	Both formulae: ↑ Short-term memory (Novel Object Recognition and Y-Maze tests) ↓ Beta-Amyloid (Aβ1–42) ↑ BDNF ↓ p-Tau ↓ Degeneration of the hippocampal tissue
Nakashima et al. [1]	Transgenic DAL101 Mice ♂/♀ (n = 15)	*Parachlorella beyerinckii* CK-5	Dosage: 5% Diet: Standard Duration: 70 weeks	Mouse Water Maze test: ↑ Time required to reach the platform Novel Object Recognition test: ↑ ability ↓ 4-HNE-positive cells in the hippocampal dentate gyrus ↓ GFAP positive cells in the hippocampal CA1 region =Number of anti-Iba1 positive cells in the hippocampus and CA1 region
Chen et al. [34]	C57BL/6 Mice ♂ (n = 30)	*C. pyrenoidosa* hot water extract—polysaccharide fraction	Dosage: 100, 200 g/kg/day Diet: Standard Duration: 19 days	*C. pyrenoidosa* (200 mg/kg/day): Pole test: ↓ Landing time Gait test: ↑ Stride length distance ↑ dopamine, DOPAC, and HVA ↑ Striatal and nigral TH ↑ TH and ↓ Emr1 mRNA expression
Abdel- Rahman et al. [29]	Swiss albino Mice ♀ (n = 80)	- *C. vulgaris* - Nicotine (100 µg/mL/kg BW in 2% saccharin solution)	Dosage: 100 mg/kg Diet: Standard Duration: 40 days (28 days before tumor induction + 12 days after tumor induction)	*C. vulgaris*: ↑ Swimming performance test =GABA, dopamine, serotonin, and AchE ↑ Bcl-2 and ↓ Caspase 3 in cerebral tissue *C. vulgaris* + Nicotine: ↑ Swimming performance test ↓ GABA, dopamine, serotonin, and AchE ↑ Bcl-2 and ↓ Caspase 3 in cerebral tissue
Wang et al. [33]	ICR Mice ♂ (n = 30)	*C. pyrenoidosa* peptides (1–3 kDa and 3–10 kDa)	Dosage: 100 mg/kg Diets: Standard Duration: 14 days	Mouse Water Maze test: ↑ Efficiency to find the target quadrant; ↓ The latency for searching the hidden platform ↑ Cell density in the hippocampus ↓ Lacunar infarction and cell loss

↑: increased; ↓: reduced; AchE: Acetyl choline esterase; ACTH: Adrenocorticotropic hormone; BDNF: Brain-Derived Neurotrophic Factor; Bcl-2: B-cell Lymphoma 2; CREB: cAMP Response Element-Binding Protein; DOPAC: Dihydroxyphenylacetic acid; GABA: Gamma-aminobutyric acid; GFAP: anti-glial fibrillary acidic protein; hnCRF: Heteronuclear RNA for corticotropin release factor; HVA: Homovanillic acid; TH: Tyrosine hydroxylase; TrKB: Tropomyosin receptor kinase B; 4-HNE: 4-hydroxy-2-nonenal.

**Table 5:** Clinical trials in humans examining the effects of *Chlorella* administration on the brain.

Author (Year)	Subjects	*Chlorella* Formulae	Study Design	Brain-Related Outcomes
Miyazawa et al. [31]	Human ♂/♀ (n = 12)	*C. pyrenoidosa*	Dosage: 8 g/day Diet: Not reported Duration: 8 weeks	↑ Lutein and β-Cryptoxanthin in erythrocytes ↓ Erythrocyte PLOOH
Panahi et al. [3]	Humans with major depressive disorders. ♂/♀ (n = 125)	*C. vulgaris* extract	Dosage: 1800 mg/day Diet: Not reported Duration: 6 weeks	Beck Depression Inventory II test: ↓ Total score; ↓ Physical and cognitive subscale; ↑ Affective subscale Hospital Anxiety Depression Scale: ↓ Total score; ↓ Anxiety and depression subscale

↑: increased; ↓: reduced; PLOOH: Phospholipid Hydroperoxide.

## Data Availability

No new data were created or analyzed in this study.

## Supplementary Materials

The following supporting information can be downloaded at: https://www.mdpi.com/article/10.3390/nu18122014/s1, Table S1. Studies excluded after full-text review and reasons for exclusion.
