# Supplementary material for: Effects of the Edible Microalga Chlorella on Gut Microbiota and on Brain Health: Current Evidence and Emerging Links
Source: Nutrients. 2026 Jun 21;18(12):2014. doi: 10.3390/nu18122014 (PMC13306205; doi:10.3390/nu18122014)
Supplement: Supplementary file 1 [file nutrients-18-02014-s001.zip › nutrients-4293025-supplementary.pdf]

Table S1. Studies excluded after full-text review and reasons for exclusion

No.	Author/Year	Study Title	Reason for Exclusion
1	Qiu et al. 2022	Metabolomics and biochemical insights on the regulation of aging-related diabetes by a low-molecular-weight polysaccharide from green microalga Chlorella pyrenoidosa.	Wrong outcome
2	Aizzat et al. 2010	Modulation of oxidative stress by Chlorella vulgaris in streptozotocin (STZ) induced diabetic Sprague-Dawley rats.	Wrong outcome
3	Queiroz et al. 2011	Chlorella vulgaris restores bone marrow cellularity and cytokine production in lead-exposed mice.	Wrong outcome
4	Hueng et al., 2023	Antidiabetic potential of Chlorella pyrenoidosa functional formulations in streptozocin-induced type 2 diabetic mice	Wrong outcome
5	Xiong et al. 2022	Anti-Diabetic Potential of Chlorella Pyrenoidosa-Based Mixture and its Regulation of Gut Microbiota	Other formulation
6	Farag et al. 2023	Benefits of Chlorella vulgaris against Cadmium Chloride-Induced Hepatic and Renal Toxicities via Restoring the Cellular Redox Homeostasis and Modulating Nrf2 and NF-KB Pathways in Male Rats	Wrong outcome
7	Hidalgo-Lucas et al. 2016	Benefits of Preventive Administration of Chlorella sp. on Visceral Pain and Cystitis Induced by a Single Administration of Cyclophosphamide in Female Wistar Rat	Wrong outcome
8	Ishiguro et al. 2017	Exopolysaccharides extracted from Parachlorella kessleri inhibit colon carcinoma growth in mice via stimulation of host antitumor immune responses	Other formulation
9	Shih et al. 2013	Chlorella 11-peptide inhibits the production of macrophage-induced adhesion molecules and reduces endothelin-1 expression and endothelial permeability	In vitro
10	Cherng et al. 2010	Beneficial effects of Chlorella-11 peptide on blocking LPS-induced macrophage activation and alleviating thermal injury-induced inflammation in rats	Wrong outcome
11	He et al. 2022	Spirulina compounds show hypoglycemic activity and intestinal flora regulation in type 2 diabetes mellitus mice	Other formulation
12	Plakida et al. 2020	The Effect of the Aqueous Suspension of Chlorella Vulgaris on Functional Systems in Healthy People	Wrong outcome
13	Renju et al. 2013	Anti-inflammatory activity of lycopene isolated from Chlorella marina on Type II Collagen induced arthritis in Sprague Dawley rats	Other formulation
14	Qi et al. 2018	α-Glucosidase Inhibitory Activities of Lutein and Zeaxanthin Purified from Green Alga Chlorella ellipsoidea	In vitro
15	Itakura et al. 2015	Chlorella ingestion suppresses resistin gene expression in peripheral blood cells of borderline diabetics.	Wrong outcome
16	Bogdanova et al. 2018	Biochemical and hematological composition of blood of cattle fed with Chlorella	Other animal species
17	Furbeyre et al. 2018	Effects of oral supplementation with Spirulina and Chlorella on growth and digestive health in piglets around weaning	Other animal species
18	Ishiguro et al. 2022	A Water Extract from Chlorella sorokiniana Cell Walls Stimulates Growth of Bone Marrow Cells and Splenocytes.	In vitro
19	Kim et al. 2023	Effect of Dietary Chlorella vulgaris or Tetradesmus obliquus on Laying Performance and Intestinal Immune	Other animal species

No.	Author/Year	Study Title	Reason for Exclusion
		Cell Parameters	
20	Janczyk et al. 2009	Microbial community composition of the crop and ceca contents of laying hens fed diets supplemented with Chlorella vulgaris.	Other animal species
21	Chou et al. 2022	Potential antidepressant effects of a dietary supplement from the chlorella and lion's mane mushroom complex in aged SAMP8 mice	Other formulation
22	Horii et al. 2017	Effects of Chlorella Pyrenoidosa on spleinc sympathetic nerve activity and tumors induced by human colon cancer cells	In vitro
23	Bañares et al., 2025	Modulation of Gut Microbiota and Short-Chain Fatty Acid Production by Simulated Gastrointestinal Digests from Microalga Chlorella vulgaris	In vitro
24	Wang et al., 2025	Unraveling the mechanisms of propofol-induced psychological dependence: a multi-omics approach linked to gut microbiota in hippocampal function	Other formulation
25	Jin et al., 2025	Nose-to-Brain Delivery of Chlorella vulgaris Extracellular Vesicles for Antidepressant Effects	Other formulation
26	Diaz et al., 2025	Chlorella vulgaris Supplementation Attenuates Lead Accumulation, Oxidative Stress, and Memory Impairment	Wrong outcome
